# Celebrating 10 Years of Research Excellence at the *Canadian Journal of Pain*

**DOI:** 10.1080/24740527.2026.2703527

**Published:** 2026-09-01

**Authors:** Joel Katz, Anna Waisman, Heather Lumsden-Ruegg

**Affiliations:** Editor-in-Chief, Canadian Journal of Pain; Editorial Manager, Canadian Journal of Pain; Editorial Assistant, Canadian Journal of Pain

This year marks the 10 year anniversary of the inaugural issue of *The Canadian Journal of Pain*, the official journal of the Canadian Pain Society. With an internationally recognized Editorial Board of more than 50 members, in 10 short years, the *Journal* has established itself as a premiere, open access forum for advancing knowledge across the full spectrum of pain research. We publish high quality scholarship spanning basic science, multidisciplinary clinical research, epidemiology, knowledge implementation, health policy, and health systems research. Our aim is to advance the understanding and treatment of pain by providing clinicians, scientists, and healthcare professionals with an interdisciplinary platform for sharing innovative research and emerging developments in the field, including chronic and neuropathic pain.

Year after year, manuscript submissions have increased in number and quality, reflecting the confidence that authors place in the *Journal* as a venue for disseminating their research. The *Journal’s* readership continues to expand, with more than 157,000 article downloads in 2025 supported by growing visibility thanks in part to new promotional initiatives that help broaden the reach of the work we publish. The *Journal* is indexed in the world’s major bibliographic databases, including Elsevier’s Scopus and Clarivate’s Emerging Sources Citation Index. The most recent bibliometrics for 2025 showcase the progress the *Journal* has made, achieving a Scopus CiteScore of 4.9, ranking 35^th^ out of the 147 journals in the Anesthesiology and Pain Medicine category. Similarly, the 2025 Clarivate Impact Factor increased to 3.2, placing it 117^th^ out of the 296 journals listed in the Clinical Neurology category.

The *Journal’s* success is also reflected in the increasingly global nature of our contributor community. We receive submissions from researchers around the world and are supported by the enthusiastic and engaged members of the Editorial Board. Board members serve as ambassadors for the *Journal*, promoting it within their professional communities, contributing their own scholarship, encouraging submissions from colleagues, and representing the *Journal* at scientific meetings and conferences worldwide.

Spearheaded by members of the *Journal’s* Editorial Board, we have published several Special Issues and have various ongoing Article Collections which explore a wide range of topics, including qualitative methods in pain research, developmental perspectives on the transition from acute to chronic postsurgical pain, social and health inequities in chronic pain, digital health and pain management, knowledge mobilization and implementation science, and phantom limb pain. These collections have highlighted emerging areas of investigation and helped bring together diverse perspectives from across the international pain community.

Several other highlights of the *Journal’s* activities deserve mention. All published article abstracts appear in French and English and authors have the option to submit and publish their manuscripts in French. Each year, the *Journal* publishes the abstracts of posters and symposia accepted for presentation at the annual meeting of the Canadian Pain Society, providing authors with a timely, citable record of their work. A popular focal point for the *Journal* features the annual Ronald Melzack Paper of the Year Award, established in honor of the Canadian pioneer of pain research and management who co-developed the Gate Control Theory of pain, created the McGill Pain Questionnaire, and proposed the Neuromatrix Theory of pain. The award recognizes outstanding, timely research published in the *Journal* that significantly contributes to fundamental or applied pain research. The award is bestowed upon the authors yearly at the Canadian Pain Society’s annual scientific meeting. Emphasizing the field’s commitment to advancing important initiatives in pain research and management, the *Journal’s* editor has joined the editors of other pain and anesthesiology journals in committing to foster the trustworthiness of pain research, maintain rigorous standards, ethical conduct, and open dialogue^1^ and promote inclusion, diversity, and equity in pain science.^2^ A final highlight of note deserves mention. Two years ago, at the recommendation of Dr. Gabrielle Pagé, and under her stewardship, the *Journal* launched the Editorial Review Mentorship Program designed to train the next generation of scientific reviewers. Fellows shadow seasoned Editorial Board members, review submitted manuscripts, and gain critical-thinking and communication skills in pain research and clinical care.

As we celebrate the 10-year anniversary milestone, we also look toward the *Journal’s* future. The landscape of scholarly publishing is evolving rapidly, and one of the most significant developments is the emergence of large language models (LLMs) and artificial intelligence (AI) tools. These technologies have the potential to enhance many aspects of the publication process, from language editing and translation to manuscript preparation, information organization, and editorial workflow. They may also help improve accessibility for authors whose first language is not English and reduce barriers to scientific communication.

As these technologies become more widely used, publishers, authors, and reviewers will need clear expectations for the integration of AI into scholarly publishing. A recent Delphi consensus developed by editors-in-chief of anesthesiology and pain medicine journals provides guidance on the responsible use of LLMs in academic publishing.^3^ The consensus recognizes that LLMs can be valuable tools for authors, reviewers, and editors when used appropriately and transparently. However, it emphasizes that human oversight remains essential. The consensus recommends full disclosure of LLM use and cautions against employing these tools to generate original ideas, data, references, conclusions, entire manuscripts, editorial decisions, or peer-review reports. Concerns regarding confidentiality, research integrity, and the proliferation of low-quality manuscripts underscore the need for its thoughtful implementation.

In the coming years, *The Canadian Journal of Pain* will explore ways to responsibly integrate AI technologies into our submission and editorial processes. As technology continues to advance, we remain committed to upholding the highest standards of scientific rigor and transparency while embracing innovations that can enhance the dissemination and accessibility of pain research.

The achievements of the past decade would not have been possible without the dedication of our authors, reviewers, readers, Editorial Board members, publishing team at Taylor & Francis, and the support of the Canadian Pain Society’s Board of Directors and membership. We thank all those who have contributed to the *Journal’s* success and growth. As we look ahead to the next 10 years, we will continue to support excellence in pain research, foster interdisciplinary collaboration, and publish work that strives to improve the lives of the hundreds of millions of people living with pain around the world.
